# Supplementary material for: In vivo imaging of central nervous system fluid spaces using synchrotron radiation-based micro computed tomography
Source: Nat Commun. 2026 May 2;17:5959. doi: 10.1038/s41467-026-71835-9 (PMC13342594; doi:10.1038/s41467-026-71835-9)
Supplement: Supplementary file 1 — Supplementary Information [file 41467_2026_71835_MOESM1_ESM.pdf]

Supplementary information

Supplementary figures

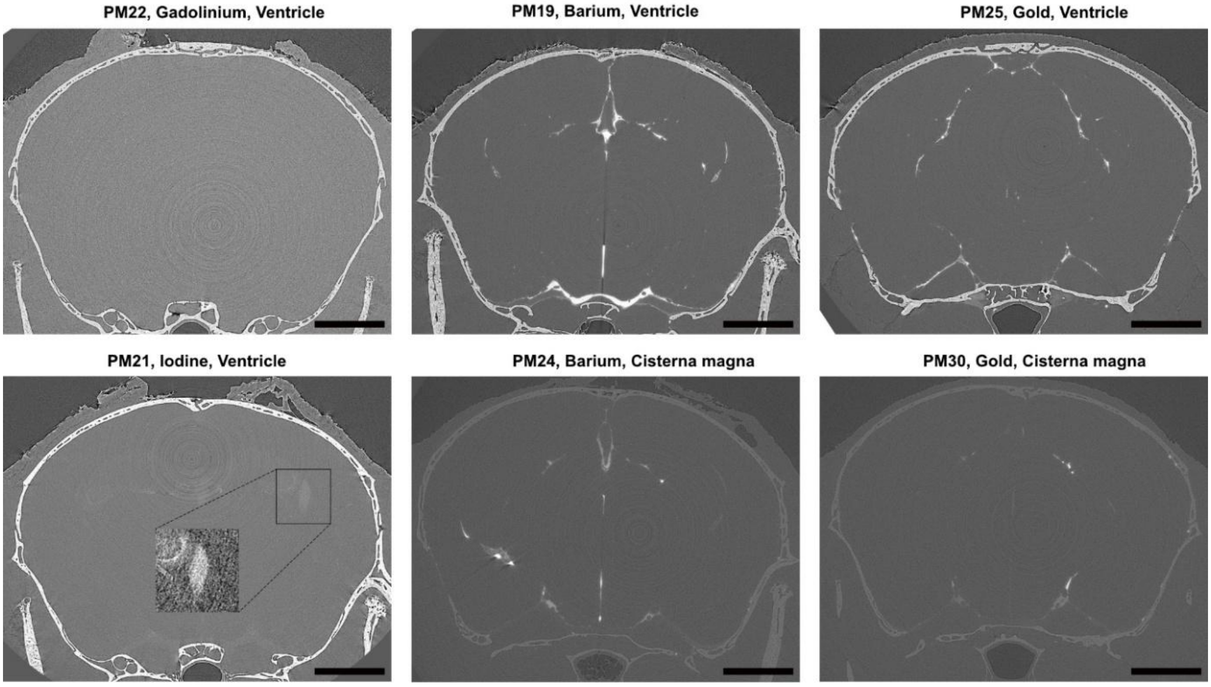

Supplementary Figure 1. Representative *post-mortem* images for contrast agent screening acquired at ID17. Images illustrate the range of outcomes in contrast distribution following either ventricular or cisterna magna injections. Each Subject_ID is given above the corresponding image. Attenuation coefficient values in the range of [- 0.38 0.68] cm^{-1} (for PM22 and PM21) or [-0.90 2.00] cm^{-1} (for PM19, PM24, PM25 and PM30) were mapped onto an 8-bit dynamic range for better visualization in this figure. Scale bars: 2 mm.

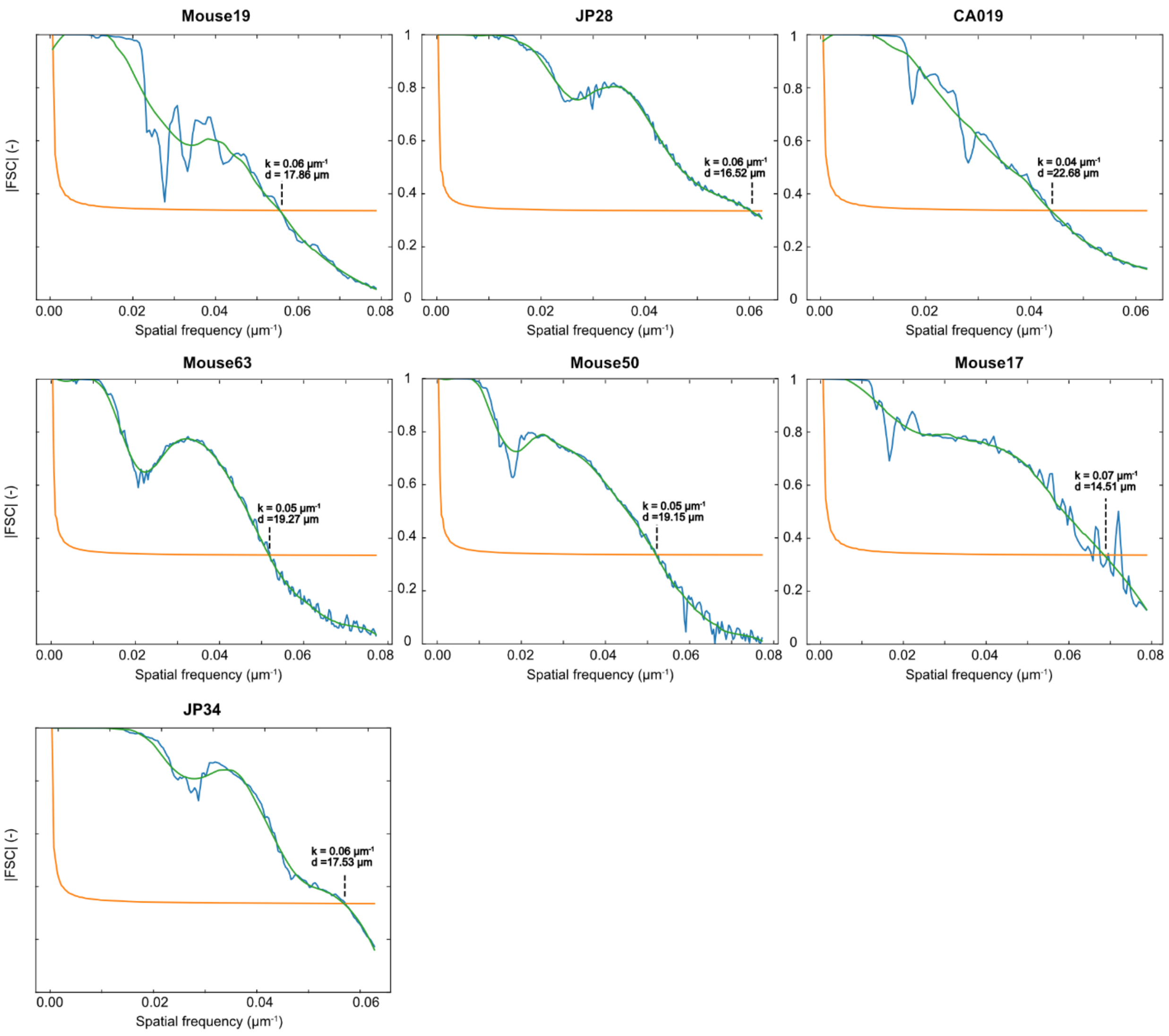

Supplementary Figure 2. Effective spatial resolution of the datasets in the manuscript. Fourier shell correlation (FSC) curves (raw in blue, smoothed in green) and 1-bit threshold curves (yellow) used to estimate spatial resolution as limited by signal-to-noise for datasets of Mouse19, Mouse63, JP28, Mouse50, CA019, Mouse17 and JP34, yielding resolutions of d = 17.9, 19.3, 16.5, 19.2, 22.7,14.5 and 17.5 µm, respectively.

Supplementary tables

SUPPLEMENTARY TABLE 1. X-RAY CONTRAST AGENTS TESTED

Subject_ID	Contrast agent	Compound type	Radiopaque element	K-edge energy (keV)	Injection site	Visual assessment[a]
PM20<br>PM26	Iohexol	Low molecular weight organic molecule	Iodine	33.2	Ventricle<br>Cisterna magna	No contrast
PM21<br>PM27	Exitron P	High molecular weight organic molecule	Iodine	33.2	Ventricle<br>Cisterna magna	Weak contrast
PM22<br>PM28	Gadodiamide	Low molecular weight chelate complex	Gadolinium	50.2	Ventricle<br>Cisterna magna	No contrast
PM23<br>PM29	GadoSpin P	High molecular weight chelate complex	Gadolinium	50.2	Ventricle<br>Cisterna magna	No contrast
PM19<br>PM25	Exitron nano 12000	Nanoparticles with hydrophilic coating	Barium	37.4	Ventricle<br>Cisterna magna	Good contrast
PM24<br>PM30	AuroVist 15 nm	Nanoparticles with hydrophilic coating	Gold	80.7	Ventricle<br>Cisterna magna	Good contrast

[a]Specimens were classified by visual assessment into three categories: no contrast (no visible intracranial contrast agent), weak contrast (contrast agent visible but contrast insufficient for *in vivo* imaging), and good contrast (sufficient contrast agent distribution and visibility for *in vivo* use).

SUPPLEMENTARY TABLE 2. EXPERIMENTAL AND IMAGING PARAMETERS

Subject_ID	Beamline	Infusion rate (µL/min)	Infused volume (µL)	Anesthesia regime[a]	Voxel size[b] (µm)	FOV[c] (mm)	Projections /scan (#)	Projection exposure time (ms)	Total scan acquisition time[d] (s)	Scan type
Mouse19	ID17	-	-	Ket/med	6.3	16.1 x 4.9	2’000	5	20	Single
JP28	BL20B2	0.25	5	Isoflurane	7.99	16.4 x 12	1’800	5	23	Single
CA019	BMIT	0.25	5	Med/mid/but	8	16.4 x 9.6	2’000	15	32	Single[e], Time series
Mouse63	ID17	0.5	2.5	Ket/med	6.45	16.5 x 4.5	2’000	4	10	Time series
Mouse50	ID17	0.2	1	Ket/med	6.45	16.5 x 4.5	2’000	4	10	Time series
Mouse17	ID17	0.5	5	Ket/med	6.3	16.1 x 4.9	60’000	5	600	Gated
JP34	BL20B2	-	-	Med/mid/but	7.92	16.2 x 11.9	1’800	4	22	Time series

[a]Anesthesia regime used during imaging. Analgesia and anesthesia induction regime are specified in the methods section and the FABRIC4 Portal. [b]Effective voxel size resulting from beamline-specific magnification optics and camera pixel size of 6.5 µm. [c]Field of view (FOV): horizontal x vertical. [d]Total scan acquisition time refers to total exposure and overhead time per scan, but excludes time needed to acquire reference images and to return the rotation stage to its original position. [e]A single scan was taken for the live state of peri-mortem imaging.

Overview of animals and experiments

Supplementary Table 2 summarizes imaging parameters and mice reported on in the manuscript, including animal identifier (Subject_ID), beamline, contrast agent infusion rate (µL/min), infused contrast agent volume (µL), anesthesia regime, voxel size (µm), field of view (mm), projections per scan (#), projection exposure time (ms), total acquisition time (s) and scan type (single, time series, or gated). For additional information on each mouse, please refer to the FABRIC4 portal.

Selection of contrast agent

Six different types of commercially available X-ray contrast agents for preclinical angiography were evaluated for their performance in imaging cerebrospinal fluid spaces in the mouse brain. Contrast agents were injected *in vivo*, and the animals were imaged post-mortem at the Biomedical Beamline ID17 of the European Synchrotron Radiation Facility (ESRF).

Methods

C57BL/6J mice (strain code 632) were supplied by Charles River Laboratoire France. To ensure proper acclimatization, the animals were housed at the beamline's animal facility for at least one week before the experiments. All procedures adhered to the European guidelines for animal experimentation (2010/63/EU). The experimental protocols were reviewed and approved by the responsible French ethics committee Comité d'éthique en expérimentation animale de l'ESRF (ETHAX), approval number APAFIS #30913-2021040211343677 v1.

12 female mice (age: 12 weeks; body weight range: 24-26 g) were first injected subcutaneously with buprenorphine (0.1 mg/kg) for analgesia. Anesthesia was induced 30 min after onset of analgesia via intraperitoneal injection of ketamine (73 mg/kg) and medetomidine (0.18 mg/kg). The anesthesia depth was monitored by testing reflexes, and additional injections of the same anesthetic mixture were given as needed. Eye ointment was applied, and the skull, neck, and upper thoracic region of the mouse were shaved to avoid imaging artifacts.

Before injection, the contrast agent to be tested (Supplementary Table 1) was carefully loaded into either a 34G/9.53 mm needle attached to a 10 µL Hamilton syringe (for intra-cerebroventricular injection) or into a cannula tube implant assembled with a Hamilton removable needle compression fitting (1 mm) attached to a 10 µL Hamilton syringe (for intra-cisterna magna injection). If bubbles were present in the syringe, the contrast agent was expelled completely and newly filled into the syringe.

Intra-cerebroventricular injection of contrast agent

The animal was carefully positioned in a stereotactic frame on a warming pad. Blunt, non-perforating ear bars were used to position the animal, paying attention that the skull was aligned in a completely horizontal position. Core body temperature of the animal was monitored and maintained stable with aid of a rectal thermometer, thermal rescue blanket and heating pad. After the re-application of eye ointment and verification of sufficient anesthesia depth, a rostro-caudal skin incision was made to expose the skull. Excess periosteum from the bone was removed and bregma was identified. A small hole of about 1 mm diameter was drilled through the parietal bone at the injection coordinates, which were 0.95 mm lateral and 0.22 mm caudal of the bregma. The tip of the 34G Hamilton syringe needle was lowered into the hole to 2.35 mm depth, and 5 µl of contrast agent

was injected at a rate of 1 µl/min over 5 min using a syringe pump. Following the injection, the needle was slowly removed after a 2 min waiting period, the skin was placed back over the injection site and sealed with tissue adhesive (e.g., Vetbond®).

Cisterna magna (CM) injection

Monitoring and maintenance of the animal's core body temperature was done as described above. The animal was placed in prone position on a custom-made stereotactic platform and blunt, non-perforating ear bars were placed. Special care was taken to ensure that the skull was in a level position. The head of the mouse was then bent downwards at an angle of 90°.

A rostro-caudal incision was made into the skin at the back of the neck. The trapezius muscle was split along the midline. The long back and short neck muscle groups were then locally disconnected and bluntly prepared until the greyish atlanto-occipital membrane became visible. This membrane was pierced with a 26G needle and a 30G cannula-tube implant was quickly inserted into the cisterna magna and sealed with a drop of cyanoacrylate glue. The implant was connected to the infusion pump. A maximum of 10 µl cyanoacrylate accelerator was applied to the bonding surfaces to reduce the curing time. This required a waiting period of about 2 min. The head of the mouse was repositioned to level position. The skin was placed back over the neck and closed with tissue adhesive (e.g., Vetbond®). 5 µl of contrast agent was injected at a rate of 1 µl/min over 5 min.

After 30 min, the mice were euthanized using an overdose of anesthesia (ketamine (160 mg/kg) and medetomidine (0.4 mg/kg)), then fixed and stored in 4 % formaldehyde for 3 days. Before imaging, the bodies were briefly dabbed with laboratory tissue paper to remove excess liquid and mounted into a customized animal holder before being transferred to the experimental hutch of the ID17 beamline of ESRF, where they were imaged using a monochromatic beam at photon energies slightly above the K-edges of the respective contrast agents listed in Supplementary Table 1. Before reaching the monochromator, the polychromatic beam passed through a fixed filter stack of 0.8 mm thick graphite, one 0.5 mm aluminum sheet, and a second aluminum sheet of 2.0 mm thickness. A total of 4000 radiographs over a rotation range of 360° were acquired with a pco.edge 5.5 camera, coupled to serial Hasselblad 100 mm f/2.2 and Hasselblad 210 mm f/4 lenses, and a 250 µm LuAG:Ce scintillator, resulting in an effective pixel size of 3.1 µm[1]. The field of view was limited to 2560 × 2019 (7.9 mm × 6.3 mm) by the vertical extent of the beam. Radiographs were recorded with 100 ms exposure time and 1 ms overhead time. Acquisition time per scan was 6 min 44 s. The specimen-to-detector distance was 0.3 m.

Radiographs were reconstructed with a filtered back-projection algorithm implemented in PyHST2 software[2]. For mouse PM21, a 2D median filter with radius 5 pixels was employed to obtain the inset in Supplementary Figure 1.

Results

For a contrast agent to be classified as suitable for *in vivo* experiments, it had to demonstrate sufficient distribution within the CSF space and provide adequate contrast to clearly visualize relevant anatomical structures, such as ventricles or subarachnoid space. Imaging post-mortem provided substantially more favorable conditions than imaging *in vivo*, as the specimens were static, eliminating motion artifacts that could have impacted the evaluation of contrast or recognition of smaller anatomic features. It also allowed for longer exposure times for higher signal-to-noise ratios.

Based on visual assessment, the specimens containing small molecular weight iodine or gadolinium-based contrast agents (0.8 kDa iohexol and 0.6 kDa gadodiamide) provided no visible intracranial contrast. PM22 is shown as a representative example of this category in Supplementary Figure 1. Small molecular weight contrast agents are known to rapidly extravasate when used in angiography. The lack of contrast may be explained by diffusion of the contrast agent out of the CSF spaces into surrounding tissue after injection.

In specimen PM21 injected with high molecular weight iodine-based ExiTron P contrast agent, structures such as the ventricles were difficult to discern and the specimen was classified as having weak contrast (Supplementary Figure 1, Supplementary Table 1). This may be explained by more limited diffusion of the 20 kDa contrast agent compared to the 0.6 or 0.8 kDa small molecular weight contrast agents. The level of contrast was considered insufficient for the envisioned *in vivo* experiments, where much shorter exposure times well below the cardiac cycle length of mice would be employed. While it is likely that the concentration of the contrast agent dropped post-mortem during the 3 days of immersion in formaldehyde – meaning that the contrast agent could have had higher contrast if imaged shortly after injection in an *in vivo* imaging experiment – confirming this finding would have required another dedicated beamtime experiment. We also note that the ExiTron P we purchased and tested in 2020 was a different compound than the one currently sold under same name (as of July 2025). Instead of a 20 kDa linear polymer, the current version is a two orders of magnitude larger polymeric capsule with 290 nm hydrodynamic diameter, which we expect to yield results more in line with the nanoparticle-based contrast agents.

The high molecular weight gadolinium chelate complex-based GadoSpin P did not show visible contrast (data not shown). Due to its size of 200 kDa, diffusion out of the CSF spaces would not explain the lower contrast compared to ExiTron P. The lack of visible contrast may instead be explained by the lower concentration of 4 mg Gd/ml of the radiopaque compound in GadoSpin P compared to the concentration of 120 mg I/ml in ExiTron P.

Both gold and barium nanoparticle-based contrast agents were classified as having good contrast. The barium-based contrast agent ExiTron nano 12000 with 110 nm diameter nanoparticles (PM19, PM25) showed the most even distribution and highest signal intensity throughout the CSF space. The gold-based contrast agent AuroVist with 15 nm diameter particles (PM24, PM30) also provided good contrast, but presented with more agglomerates. This was observed in both ventricular (PM19 against PM24) and cisterna magna (PM25 against PM30) injections (Supplementary Figure 1). Consequently, the barium-based ExiTron nano 12000 was chosen for our *in vivo* imaging experiments.

References

1. Mittone, A., Manakov, I., Broche, L., Jarnias, C., Coan, P. & Bravin, A. Characterization of a sCMOS-based high-resolution imaging system. J. Synchrotron Rad 24, 1226-36 (2017).
2. Mirone, A., Gouillart, E., Brun, E., Tafforeau, P. & Kieffer, J. PyHST2: an hybrid distributed code for high speed tomographic reconstruction with iterative reconstruction and a priori knowledge capabilities. *Nucl Instrum Methods Phys Res B* 324, 41–48 (2013).
