# Supplementary material for: In vivo imaging of central nervous system fluid spaces using synchrotron radiation-based micro computed tomography
Source: Nat Commun. 2026 May 2;17:5959. doi: 10.1038/s41467-026-71835-9 (PMC13342594; doi:10.1038/s41467-026-71835-9)
Supplement: Supplementary file 2 — Description of Additional Supplementary Files [file 41467_2026_71835_MOESM2_ESM.pdf]

Description of Additional Supplementary Files

Supplementary Movie 1. Peri-mortem shrinkage of contrast agent-free ventricular spaces in a mouse euthanized with an overdose of anesthetics (BL20B2, Subject_ID: JP34). The movie shows a time-lapse of 60 reconstructed and registered coronal slices depicting the lateral ventricular region where contrast agent would be infused for contrast-enhanced imaging. Time between frames is 40 s. The video is rendered at 4 frames per second, corresponding to 160× speed. Recording begins immediately after anesthetic overdose. From 14 to 19 min (time points 20 to 29), movement artifacts are visible across the entire image, caused by body movement during unconscious deep final breaths before respiratory arrest. Following this period, progressive shrinkage of the ventricles can be observed, accompanied by a more pronounced choroid plexus movement compared to the live period at the beginning of the movie. Scale bar: 1 mm. Movie was downsampled 2×. Full resolution movie is available on Zenodo: https://doi.org/10.5281/zenodo.13773080

Supplementary Movie 2: Mapping spatiotemporal solute distribution throughout the cranial CSF space following intra-cerebroventricular infusion – coronal reconstructions (ID17, Subject_ID: Mouse63). The movie shows reconstructed and registered coronal slices of a plane intersecting the right lateral ventricle infusion site, see Fig. 4d (left). The time series consisted of 100 scans acquired every 30 s and shown as timelapse at 120× speed. By the 19th time point (9 min), the peak of attenuation coefficient in the right lateral ventricle was recorded as 7.5 cm^{-1}, see Fig. 4c (left). At this time, the contrast agent had filled the lateral ventricles and the third ventricle. Subsequently, contrast agent concentration in the right ventricle decreased until the end of the time series, with final measured values at the 100th time point of 1.5, 1.0 and 1.0 cm^{-1} for the right lateral ventricle, the third ventricle and the left lateral ventricle, respectively. The dynamic profile depicted in this video is quantitatively represented in Fig. 4c (left), which shows the temporal evolution of the attenuation coefficients for selected regions of interest. Scale bar: 1 mm. Movie was downsampled 2×. Full resolution movie is available on Zenodo: https://doi.org/10.5281/zenodo.13773080

Supplementary Movie 3: Mapping spatiotemporal solute distribution throughout the cranial CSF space following intra-cerebroventricular infusion – projections (ID17, Subject_ID: Mouse63). The movie shows non-registered radiographs from the starting position of the rotation stage at 0°.

Acquisitions were made in 30 s-intervals and are shown as timelapse at 120× speed. In this view, the infusion cannula delivering the contrast agent, as well as contrast agent distribution throughout the lateral ventricles can be observed. After the 20th time point (9.5 min), the contrast agent in the infusion cannula is slowly pushed back into the infusion syringe due to backpressure. To counter this, a maintenance infusion at very low infusion rates after actual contrast agent delivery was introduced in later experiments. Scale bar: 1 mm. Movie was downsampled 2×. Full resolution movie is available on Zenodo: https://doi.org/10.5281/zenodo.13773080

Supplementary Movie 4: Mapping spatiotemporal solute distribution throughout the cranial CSF space following intra-cisterna magna infusion (ID17, Subject_ID: Mouse50). The movie displays reconstructed and registered coronal slices of a plane intersecting the tip of the infusion cannula. The time series consisted of 50 scans each acquired every 30 s, shown at 120× speed. By the 7th time point (3 min), the maximum attenuation coefficient value in the cisterna magna was recorded as 4.5 cm^{-1}, see Fig. 4c (right). At this time, the contrast agent was also entering the subarachnoid space on either side of the cisterna magna. Subsequently, contrast agent concentration decreased to below 1.0 cm^{-1} within 3 min. The dynamics depicted in this video are quantitatively represented in Fig. 4c (right), which shows the temporal evolution of the mean attenuation coefficient for selected regions of interest. Scale bar: 1 mm. Movie was downsampled 2×. Full resolution movie is available on Zenodo: https://doi.org/10.5281/zenodo.13773080

Supplementary Movie 5: Quantifying extracranial tissue motion (ID17, Subject_ID: Mouse17). Movie showing movement of the nasopharynx in coronal slices reconstructed with retrospective cardiac gating. Each frame represents a different phase in the cardiac cycle, with 10 ms interval between frames, shown at 1/25× speed. The most prominent movement can be observed during the first half of the movie. Scale bar: 1 mm.

Supplementary Movie 6: Quantifying intracranial tissue motion (ID17, Subject_ID: CA019). Movie of orthogonal slices showing the left lateral ventricle after contrast agent arrival (13 min) from the infused right ventricle. Slow, non-periodic movement of the choroid plexus can be observed. Images were acquired every 1 min and are shown as timelapse at 240× speed, except between minutes 30 and 32 (time points 31 and 33), which are 2 min apart. Scale bar: 1 mm.
