# Supplementary material for: In vivo imaging of central nervous system fluid spaces using synchrotron radiation-based micro computed tomography
Source: Nat Commun. 2026 May 2;17:5959. doi: 10.1038/s41467-026-71835-9 (PMC13342594; doi:10.1038/s41467-026-71835-9)
Supplement: Supplementary file 10 — Transparent Peer Review file [file 41467_2026_71835_MOESM10_ESM.pdf]

nature portfolio

Peer Review File

In vivo imaging of central nervous system fluid spaces using synchrotron radiation-based micro computed tomography

Corresponding Author: Professor Vartan Kurtcuoglu

This manuscript has been previously reviewed at another journal. The manuscript was considered suitable for publication without further review at Nature Communications.

Open Access This Peer Review File is licensed under a Creative Commons Attribution 4.0 International License, which permits use, sharing, adaptation, distribution and reproduction in any medium or format, as long as you give appropriate credit to the original author(s) and the source, provide a link to the Creative Commons license, and indicate if changes were made.
In cases where reviewers are anonymous, credit should be given to 'Anonymous Referee' and the source.
The images or other third party material in this Peer Review File are included in the article's Creative Commons license, unless indicated otherwise in a credit line to the material. If material is not included in the article's Creative Commons license and your intended use is not permitted by statutory regulation or exceeds the permitted use, you will need to obtain permission directly from the copyright holder.
To view a copy of this license, visit https://creativecommons.org/licenses/by/4.0/
